# The Influence of Menstrual Cycle Phases on Maximal Strength Performance in Healthy Female Adults: A Systematic Review with Meta-Analysis

**DOI:** 10.3390/sports12010031

**Published:** 2024-01-12

**Authors:** Marc Niering, Nacera Wolf-Belala, Johanna Seifert, Ole Tovar, Jacqueline Coldewey, Jennifer Kuranda, Thomas Muehlbauer

**Affiliations:** 1 Institute of Biomechanics and Neurosciences, Nordic Science, 30173 Hannover, Germany; niering@nordicscience.de (M.N.); wolf-belala@nordicscience.de (N.W.-B.); seifert.johanna@mh-hannover.de (J.S.); 2 Department of Psychiatry, Social Psychiatry and Psychotherapy, Medical School Hannover, 30625 Hannover, Germany; 3 Department of Sports Science, Bielefeld University, 33615 Bielefeld, Germany; ole.tovar@uni-bielefeld.de; 4 Institute of Sport Sciences, Biosciences of Sports, University of Hildesheim, 31141 Hildesheim, Germany; coldew@uni-hildesheim.de; 5 Triagon Academy Munich, School of Sports, Psychology and Education, 85737 Ismaning, Germany; jennifer.kuranda@edu.triagon-academy.com; 6 Division of Movement and Training Sciences/Biomechanics of Sport, University of Duisburg-Essen, 45141 Essen, Germany

**Keywords:** female, menstrual cycle, maximal strength, individualization, elite athlete, physiology, performance

## Abstract

Maximal strength is a significant factor in achieving peak performance and injury prevention in athletes. In individualization strategies for the efficient development of athletes, it is necessary to consider the respective components separately. The purpose of this study was to systematically examine the effects of the different cycle phases on isometric, isokinetic, and dynamic maximum strength. A systematic literature review was conducted; databases were searched from January 1960 to September 2023. The included studies focused on the expression of maximal strength in the earlier follicular phase as well as at least one comparative phase. Of the initial 707 articles identified, 22 met the selection criteria and were included. The studies considered a total of 433 subjects. Our results revealed medium effects (weighted mean standardized mean difference (*SMD*) = 0.60; seven studies) for isometric maximal strength in favor of the late follicular phase, small effects (weighted mean *SMD* = 0.39; five studies) for isokinetic maximal strength in favor of the ovulation phase, and small effects (weighted mean *SMD* = 0.14; three studies) for dynamic maximal strength in favor of the late follicular phase. The results indicate that the early follicular phase is unfavorable for all strength classes. Peak performance in isometric strength is seen in the late follicular phase, whereas isokinetic strength peaks during ovulation. Dynamic strength is optimal in the late follicular phase.

## 1. Introduction

The participation and promotion of women at the international level in many sports disciplines has only slowly become established in recent decades, which has meant that sports-related tests have predominantly been carried out on male subjects [1]. If women were included in testing, measurements were often taken at low hormone levels to minimize potential hormonal influence [2]. Previous literature shows that training monitoring has been researched at a physiological level for the male body, and the results have been transferred to design training programs for female athletes without examining its transferability [3]. Although the influence of menstrual cycle phases encompasses many areas of physical and mental health [4], strength performance emerges as the most significant for adolescent and adult athletes [5,6].

Maximal strength is an essential component of physical fitness and biomechanics, defining the maximum force that a muscle or group of muscles can produce. This central concept can be divided into different categories based on the mode of muscle contraction and the conditions under which strength is measured. As identified by Haff et al. [7] and Zaciorskij and Kraemer [8], these categories are isometric, isokinetic, and dynamic. Isometric maximal strength is the force that a muscle or muscle group can produce without significant change in muscle length (i.e., maximal voluntary isometric contraction [MVIC]). This involves the individual exerting maximum force against a stationary object or resistance while keeping the muscle length and joint angle constant [9]. In terms of isokinetic maximal strength, it represents the peak force produced by the muscles during a contraction maintained at a constant velocity (i.e., isokinetic dynamometry) [10]. Lastly, dynamic maximal strength focuses on the force generated during the concentric and eccentric phases, which is usually determined by the maximum weight an individual can lift for one repetition through a full range of motion (i.e., one repetition maximum [1 RM]) [11].

In sports, particularly for female athletes, achieving high levels of maximal strength is not only critical for enhancing performance but also for injury prevention. Studies have shown that improved maximal strength is inversely related to injury rates, meaning that female athletes with superior maximal strength often experience fewer injuries [12,13]. Furthermore, during the rehabilitation phase following a sports injury, a robust base of maximal strength can be considered beneficial in preventing potential re-injury [14]. Consequently, variations across the cycle may pose an additional injury risk factor, highlighting the importance of maximal strength in injury prevention, particularly for female athletes.

Further examination of factors influencing maximal strength, and therefore injury prevention and performance, has revealed a variety of elements that are not sex-specific. However, for female athletes in particular, certain determinants affect maximal strength as the menstrual cycle is factored in. These include hormonal ebbs and flows, particularly of estrogen and progesterone [15,16]; muscle and tendon stiffness [17,18], which is a key component of maximal strength [19] and injury rehabilitation [20]; pain perception and therefore maximal performance capacity during strength exercises [21]; fluid dynamics within the body, particularly retention patterns, affecting muscle functionality and perceived exertion during strength training [22]; joint stability and laxity, affecting performance efficiency [23,24]; endurance metrics and fatigue resistance during resistance exercise [25,26]; and psychological components of intrinsic motivation and perceived exertion [27,28].

The menstrual cycle, as described by Janse de Jonge et al. [2], progresses from the early follicular phase (1), characterized by low levels of estrogen and progesterone, through the late follicular phase (2), with increasing levels of estrogen, to the ovulatory phase (3), characterized by high levels of estrogen and a gradual increase in progesterone, followed by the early luteal phase (4), characterized by a predominant progesterone surge, culminating in the mid-luteal phase (5), with peak levels of both estrogen and progesterone, and finally the late luteal phase (6), when both hormone levels decline, signaling the onset of menstruation.

When considering the bio- and neurophysiological foundations for the influence of cycle phases on maximal strength, the current literature primarily reports that (a) sex hormones, (b) energy metabolism, (c) neuromuscular function, and (d) body composition are significant factors; thus, these factors are highlighted below. Female sex hormones may influence muscle physiological adaptations in the areas of hypertrophy, force development, and lactate metabolism [29]. While estrogen has a neuroexcitatory effect [30] and thus influences activity potential, force production, and performance in a positive way, progesterone inhibits cortical excitability and thus represents a negative influence [30,31,32,33]. The strength-enhancing effect of estrogen suggests that skeletal muscle is an estrogen-responsive tissue [34] and that mRNA and protein levels of α-estrogen receptors in skeletal muscle are sensitive to circulating estrogen levels [35]. Furthermore, it has been shown that myosin is directly affected by the estrogen concentration [34] and that under the condition of reduced estrogen levels, as occurs during menstruation, the number of active myosin heads bound to actin is reduced. Thus, the muscle’s ability to generate force is diminished [36], and improved maximum force performance during the late follicular phase (estrogen +/progesterone −), compared to the luteal phase (estrogen −/progesterone +), can be expected, whereupon training recommendations can be derived. Another possible cause of altered strength performance is a menstrual cycle-related change in available testosterone. An acute increase in testosterone levels can enhance physical performance via improved neural activation and also improve electrophysiological and contractile properties of muscles as well as motor system function [37]. Studies show that a spike in testosterone is present during the ovulatory phase [38,39].

Another effect of altered hormone concentrations is expressed in terms of energy metabolism. During the mid-luteal phase, there is an increased reliance on lipid metabolism, while carbohydrate utilization decreases due to higher estrogen levels compared to the early follicular phase [40]. Estrogen is known to enhance fat utilization, potentially leading to a sparing effect on muscle glycogen stores. This shift in energy substrate utilization might favor endurance performance in the mid-luteal phase, as the body can conserve its limited glycogen stores and rely more on abundant fat reserves for energy production [41]. On the other hand, the late follicular phase is favorable for glucose utilization, as insulin sensitivity increases [42], which improves intensive and short-duration exercise [43]. For athletes with type 1 diabetes, it is particularly relevant to understand that insulin sensitivity is lower during the luteal phase compared to the follicular phase of the menstrual cycle [44]. This means that during the luteal phase, the body’s ability to uptake glucose, which is essential for achieving maximal strength, may be reduced. Furthermore, Justice et al. [45] described a positive correlation between increased insulin sensitivity, 1RM, and motor function.

Considering the relevance of neuromuscular function to the execution of explosive, maximal strength movements, the discharge behavior of motoneurons during fast muscle contractions is performance-determining [46]. In this context, compared to the early follicular phase, the late luteal phase shows both a higher initial firing rate of the motor units of the medial and oblique vastus medialis [47] and reduced fatigability [48].

Further temporary fluctuations in body composition during a menstrual cycle could also be an influencing factor for altered maximal strength performance. Increased body mass is associated with reduced aerobic and anaerobic endurance performance [49,50,51]. In athletes and healthy non-athletes, body mass and total body water increased from the follicular to the luteal phase [52,53]. This increase in body mass in the luteal phase could be caused by the decrease in insulin as progesterone increases, which drives appetite and food intake [54], or by fluid retention, as aldosterone, the thirst hormone, is increased in the luteal phase [55].

Recent reviews have shown very heterogeneous results [27,56,57,58,59] due to the multiple influencing factors within the cycle phases, although to the authors’ knowledge, no systematic review has explicitly examined the effects on maximal strength. This provides the rationale for this systematic review and meta-analysis, namely, to both provide initial insights into differences in maximal strength and develop further practical implications.

This systematic review and meta-analysis aims to determine whether menstrual cycle hormone fluctuation influences maximal isometric, isokinetic, and dynamic strength. Due to the bio- and neurophysiological differences identified during the menstrual cycle phases and their potential influence on maximal strength, it is hypothesized that these phases not only significantly influence the expression of maximal strength but also lead to distinct differences between the three categories. The findings of this study can enhance the understanding of female-specific physiological responses in sports and contribute significantly to the growing field of sex-specific sports medicine and training. If studies indicate a difference in performance within the menstrual cycle, increased sex-specific research would be of great importance. This could benefit female athletes, who could experience an increase in performance through cycle-based training. To be able to describe the mode of action of hormone fluctuations more accurately, the following analysis uses the six-phase model described by McNulty et al. [27].

## 2. Materials and Methods

This meta-analysis was conducted in accordance with the PRISMA guidelines [60] (Table S1). The following chapter describes in detail the individual steps of the applied methodology.

### 2.1. Literature Search

The search for appropriate literature was conducted in the PubMed, Medline, SportDiscus, and Web of Science databases using the following Boolean operators: (“menstrual cycle” AND “follicular”) AND (“isokinetic strength” OR “isometric strength” OR “maximal strength” OR “dynamic strength” OR “muscle strength” OR “muscle power”). In addition, the reference lists were searched to identify further relevant studies. Only English-language results were searched in all databases. Studies with publication dates between January 1960 and September 2023 were searched.

### 2.2. Selection Criteria

The eligibility of the studies was checked according to the PICOS scheme (population, intervention, comparator, outcome, study design) and is shown in Table 1. In this meta-analysis, women with a regular menstrual cycle were selected as the population. The intervention describes the measurement of muscle strength in a clearly defined cycle phase. The early follicular phase serves as the control group, as this can be easily verified by the onset of menstrual bleeding. This must be compared with at least one other cycle phase to draw a comparison. A variable of maximal isometric, isokinetic, or dynamic strength must be specified as an outcome. Finally, the following requirements had to be fulfilled regarding the study design: (a) within-subjects study design wherein participants were assessed in at least two cycle phases, (b) the measurements were taken on a group not taking a contraceptive pill, and (c) the strength values were presented numerically. Furthermore, the selection of studies was based on the following exclusion criteria: (a) subjects had menstrual cycle dysfunction, (b) subjects used a contraceptive pill, (c) the study did not examine maximal isometric, isokinetic, or dynamic strength, (d) no English full-text version could be located, and (e) the study was not conducted in humans.

According to Guellich and Schmidtbleicher [61], the definition of maximum strength was set as the highest force that the neuromuscular system can develop during a maximum voluntary contraction. Therefore, studies that measured muscle power or impulse were excluded.

### 2.3. Methodological Study Quality

The quality of each study was assessed using an adapted Downs and Black checklist (Table S2) according to Mc. Nulty et al. [27] and was conducted independently by two authors (OT, JC). In case of disagreement, a third author (NWB) was consulted for the majority decision. The modified list deals with five categories in which a maximum of 16 points can be scored. These points are obtained through 15 items in the categories of reporting, external validity, risk of bias, selection bias, and significance. The score obtained determined the study quality as very low (0–5 points), low (6–9), moderate (10–13), or high (14–16). The score was then used to determine the final study quality based on two additional questions. The authors made this adjustment based on the scientific recommendations of Knowles et al. [62] and Janse De Jonge et al. [2]. These explain the relevance of a standardized verification of the cycle phases. Accordingly, the first step was to answer whether the cycle phases were confirmed by blood sampling. If this question was answered in the negative, the quality of the study was lowered by one level. If the method was used, the study retained its previously determined quality status. This was followed by the question of whether a urinary ovulation test was performed to verify ovulation. The consequence of a negative answer was again the downgrading of the quality.

### 2.4. Data Extraction and Data Processing

All literature results were stored in a literature management program. Once the appropriate studies were identified, the essential data could be extracted. The selected categories are shown in Table 1, and their preparation was based on the PICOS scheme. In the studies included, various menstrual cycle phase models were utilized; therefore, a partial adjustment was made for this study. For uniform categorization, the days of the measurements were used and classified according to the aforementioned six-phase model.

This systematic review with meta-analysis considers several outcome measures, which are listed in Table 2. The included studies were screened for the outcome variables of interest. To minimize the heterogeneity between studies, we presented the preferred and alternative measures for each outcome in Table 2, considering the wide range of test procedures used in the literature. In the category of maximal isometric strength, maximal voluntary isometric contraction (MVIC) of the knee extensors was identified as the most common outcome. Regarding maximal isokinetic strength, the most relevant measure is the peak torque of knee extensors at a velocity of 60°/s, as it is the most commonly used and suggested to reveal the maximal capacity of the muscle to produce force [63,64]. For maximal dynamic strength, only three different outcomes were identified, with the 1RM half-squat set as the preferred outcome, as it is the only closed-chain exercise that shows the highest relevance in terms of practical application [11]. In one included study [65], shoulder internal rotation was preferred to external rotation and abduction as outcomes since it has the highest relevance in everyday life [66] as well as in sports [67]. If studies used other outcomes that could be assigned to one of the three categories, these were presented as alternative outcomes in Table 2.

### 2.5. Statistical Analysis

The data were analyzed using Cochrane’s Review Manager 5.4.1. To identify differences in different cycle phases, a subgroup analysis was conducted. A subgroup was created if at least three measurements were available for a cycle phase. In each subgroup analysis, the early follicular phase served as a control condition and was compared to another phase (i.e., comparator condition). The standardized mean difference (SMD) was calculated to assess differences between the control condition and the comparator conditions. A positive/negative SMD value indicates better maximal strength performance for the comparator/control condition. Further, and in accordance with Cohen [68], SMD values of 0 ≤ 0.49 indicate small effects, values of 0.50 ≤ 0.79 indicate medium effects, and values of ≥0.80 indicate large effects. *I*^2^ statistics were assessed according to the ranges given by Deeks et al. [69]. These are defined as 0–40%: trivial heterogeneity, 30–60%: moderate heterogeneity, 50–90%: substantial heterogeneity, and 75–100%: considerable heterogeneity. It is emphasized here that the assessment of the *I*^2^ statistics should not be used solely to make a statement about heterogeneity. The ranges of values given serve as a rough classification [70].

## 3. Results

### 3.1. Selection of Studies

**Figure 1:**
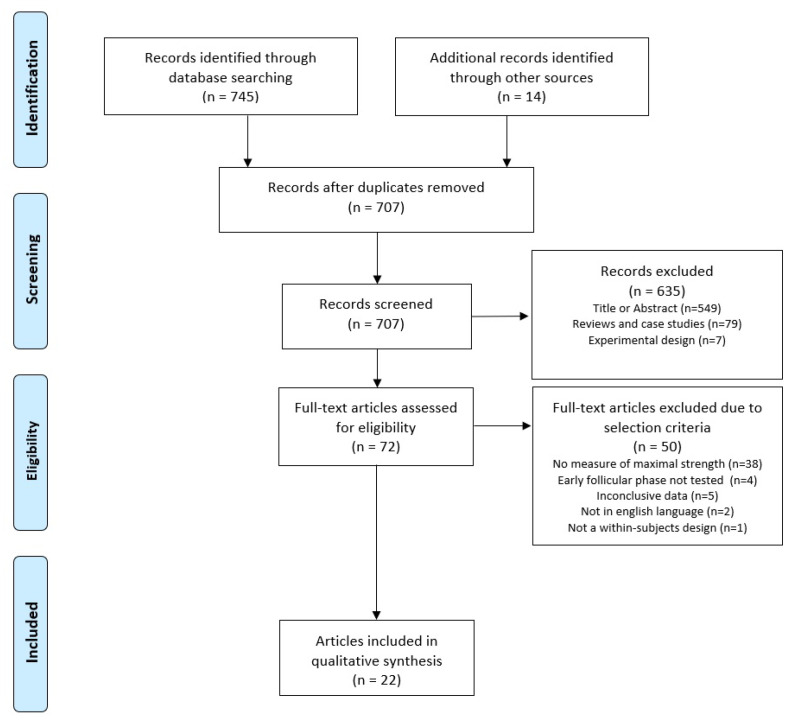
Flowchart illustrating the different phases of the literature search and study selection.

The process of the literature search is illustrated in Figure 1. The English PRISMA flow chart for systematic reviews [60] served as a template. The search yielded a total of 745 hits as well as 14 additional hits through a manual search of reference lists. After removing duplicates and excluding articles based on title or abstract as well as reviews, case studies, and articles with experimental study designs, 707 studies remained. Accordingly, seventy-two articles were subjected to a full-text search; of these articles, thirty-eight did not report a measurement of maximal strength, four did not test in the early follicular phase, five provided inclusive data, two were not written in English, and one did not use a within-subjects design.

### 3.2. Study Characteristics

Table 3 shows the characteristics of the studies analyzed. In the 22 included studies, a total of 433 subjects were tested for maximal strength performance in at least two phases of the menstrual cycle. The examinations comprised measurements taken during one to five phases of the menstrual cycle. Of the studies analyzed, all examined the early follicular phase, eleven the late follicular phase [6,28,31,32,71,72,73,74,75,76,77], twelve the ovulation phase [28,65,71,73,74,78,79,80,81,82,83,84], four the early luteal phase [28,72,73,85], and nineteen the mid-luteal phase [6,31,32,65,71,72,74,75,76,78,79,80,81,82,83,84,85,86,87]. The late luteal phase was mentioned in two studies [28,31]. Fifteen studies measured maximal isometric strength [28,32,65,72,73,74,75,77,80,81,82,83,84,85,86], eight maximal isokinetic strength [31,71,75,79,80,81,84,87], and three maximal dynamic strength [6,76,78]. Nine studies determined the menstrual cycle phase using blood samples [72,74,75,78,80,81,82,85,87], six using urine samples [6,65,71,73,83,86], three counting days [76,77,79], and one each using saliva samples [31], self-report [84], body temperature [28], or did not report the method [32].

### 3.3. Methodological Study Quality

Table S2 shows the results of the quality check using the adapted Downs and Black Checklist. A total of seven studies were rated as very low [28,32,73,76,77,79,84], eight as low [6,31,65,71,74,78,83,86], and six as moderate [72,75,80,81,82,87]. Only one of the studies was of high quality [85], and no study was of very high quality.

### 3.4. Influence of the Cycle Phases on Maximal Strength Performance

**Figure 2:**
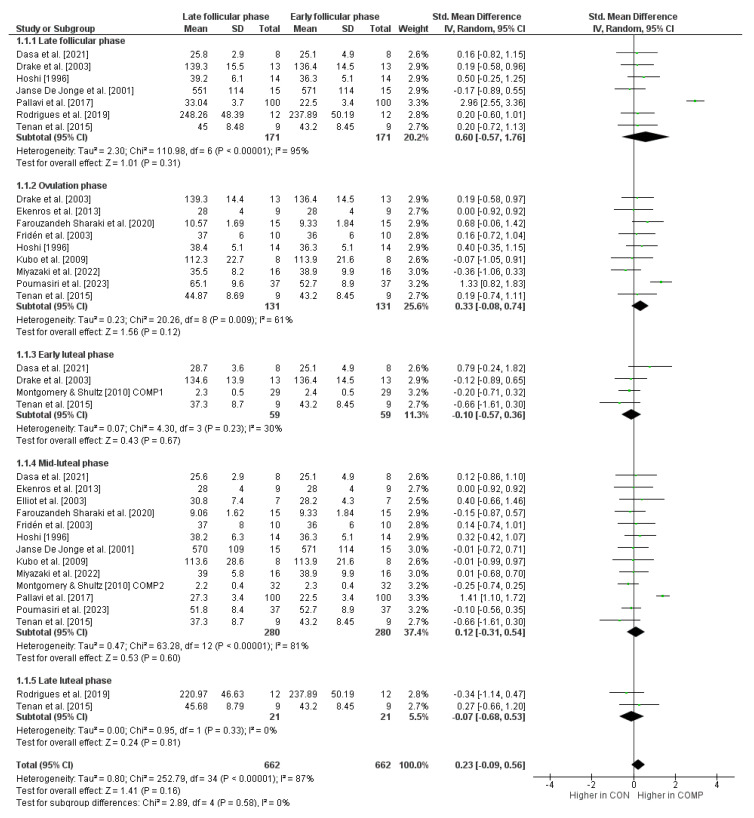
Differences in measures of maximal isometric strength performance by menstrual cycle phase [32,47,65,72,73,74,75,77,80,81,82,83,84,85,86]. *CI* = confidence interval, *COMP* = comparator condition (i.e., at least one other cycle phase like late follicular phase, etc.), *CON* = control condition (i.e., early follicular phase), *df* = degrees of freedom, *SE* = standard error, and *IV* = inverse variance.

 The maximal isometric strength performance for the early follicular phase compared to the other phases of the menstrual cycle is shown in Figure 2. The weighted mean *SMD* amounted to 0.60 when compared to the late follicular phase (*I*^2^ = 95%, Chi^2^ = 110.98, *df* = 6, *p* < 0.00001, seven studies), 0.33 for the ovulation phase (*I*^2^ = 61%, Chi^2^ = 20.26, *df* = 8, *p* = 0.009, nine studies), −0.10 for the early luteal phase (*I*^2^ = 30%, Chi^2^ = 4.30, *df* = 3, *p* = 0.23, four studies), 0.12 for the mid-luteal phase (*I*^2^ = 81%, Chi^2^ = 63.28, *df* = 12, *p* < 0.00001, thirteen studies), and −0.07 for the late luteal phase (*I*^2^ = 0%, Chi^2^ = 0.95, *df* = 1, *p* = 0.33, two studies), which is indicative of small to medium effects.

**Figure 3:**
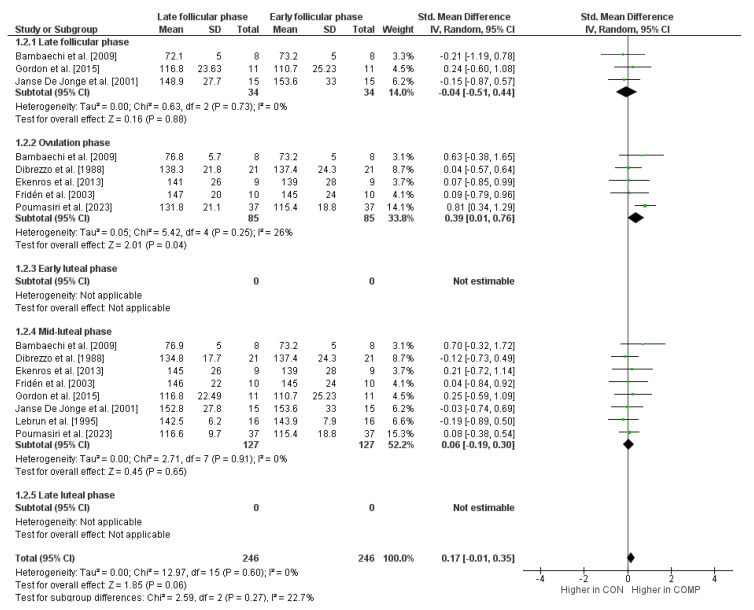
Differences in measures of maximal isokinetic strength performance by menstrual cycle phase [31,71,75,79,80,81,84,87]. *CI* = confidence interval, *COMP* = comparator condition (i.e., at least one other cycle phase like late follicular phase, etc.), *CON* = control condition (i.e., early follicular phase), *df* = degrees of freedom, *SE* = standard error, and *IV* = inverse variance.

In terms of maximal isokinetic strength performance, the comparison of the early follicular phase with the other phases of the menstrual cycle is shown in Figure 3. The weighted mean *SMD* yielded −0.04 when compared to the late follicular phase (*I*^2^ = 0%, Chi^2^ = 0.63, *df* = 2, *p* = 0.73, three studies), 0.39 for the ovulation phase (*I*^2^ = 26%, Chi^2^ = 5.42, *df* = 4, *p* = 0.25, five studies), and 0.06 for the mid-luteal phase (*I*^2^ = 0%, Chi^2^ = 2.71, *df* = 7, *p* = 0.91, eight studies), which indicates small effects. No *SMD* values were calculated for the comparison of the early follicular phase with the early and late luteal phases since our search did not identify the required minimum of two studies.

**Figure 4:**
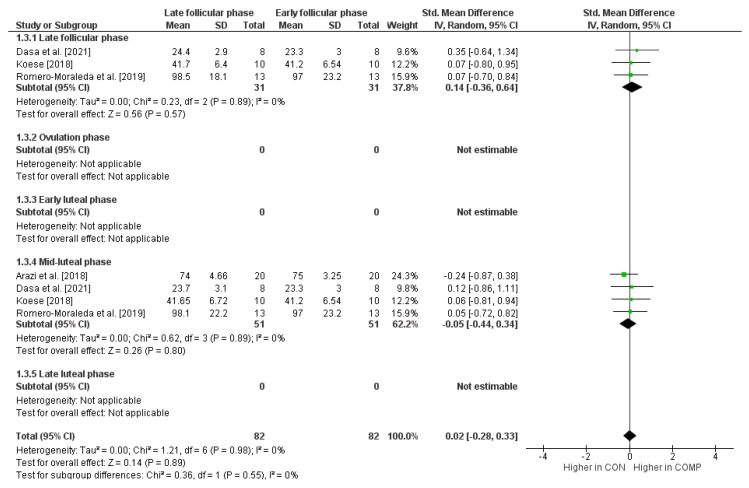
Differences in measures of maximal dynamic strength performance by menstrual cycle phase [6,72,76,78]. *CI* = confidence interval, *COMP* = comparator condition (i.e., at least one other cycle phase like late follicular phase, etc.), *CON* = control condition (i.e., early follicular phase), *df* = degrees of freedom, *SE* = standard error, and *IV* = inverse variance.

Figure 4 shows maximal dynamic strength performance for the early follicular phase compared to the other phases of the menstrual cycle. The weighted mean *SMD* amounted to 0.14 when compared to the late follicular phase (*I*^2^ = 0%, Chi^2^ = 0.23, *df* = 2, *p* = 0.89, three studies) and −0.05 for the mid-luteal phase (*I*^2^ = 0%, Chi^2^ = 0.62, *df* = 3, *p* = 0.89, four studies), which is again indicative of small effects. For the comparison of the early follicular phase with the ovulation phase, the early luteal phase, and the late luteal phase, no *SMD* values were calculated since our search did not detect a minimum of two studies.

## 4. Discussion

This systematic review and meta-analysis was conducted to determine whether menstrual cycle hormone fluctuation influences maximal isometric, isokinetic, and dynamic strength. The 22 studies analyzed, encompassing a total of 433 women, showed heterogeneous results.

Most studies (86.4%) compared several phases (two or more) of the menstrual cycle, using the early follicular phase as a control condition to account for all relevant hormonal changes. This approach included comparisons of women’s performance during the early follicular phase with that of the late follicular, ovulation, early luteal, mid-luteal, and late luteal phases. However, at the same time, the relatively small sample sizes (N = 7–100) of the studies can be criticized because of the resulting small effects. Furthermore, only three studies (13.6%) qualified for a power analysis. These studies, however, only compared a maximum of three menstrual cycle phases. All the remaining studies did not include a power analysis, and their results therefore forfeit informative value.

Nevertheless, it can be summarized that regarding maximal isometric strength, 15 studies including 334 participants found that the late follicular phase (*SMD* = 0.60) followed by the ovulation phase (*SMD* = 0.33) showed increases in outcome measures compared to the early follicular phase, while the luteal phase resulted in decreased values (*SMD* values of −0.10, 0.12, and −0.07) and therefore seemed to be a more unsuitable point of time in the menstrual cycle for isometric strength assessment. This finding is in line with the hormone fluctuation during these phases, namely, the increased level of estrogen, which with its neuroexcitatory function and strength-enhancing effect influences force production in a positive manner, as well as the decrease in progesterone, which inhibits cortical excitability and therefore displays a negative influence in activity potential and performance [30,31,32,33]. During the late follicular phase (around day 11), the estrogen level reaches its peak value, while the progesterone level remains low. Around day 14, the progesterone level starts rising again until the mid-luteal phase (around day 23). At the same time, the estrogen level is low [27]. In the subsequent mid-luteal phase, both hormones rise to a high level. Here, the increased progesterone has a negative effect on the anabolic effect of estrogen and thus inhibits its effect [88], which might be a reason for the weak results during this phase measured in the included studies. Another factor not to be forgotten is the level of testosterone, due to its performance-enhancing character and its increased availability during the ovulation phase [38].

Regarding maximal isokinetic strength, which was investigated in eight studies including 127 participants, the ovulation phase (*SMD* = 0.39) followed by the mid-luteal phase (*SMD* = 0.06) seemed to be a more appropriate point of time in the menstrual cycle for improved performance, while the late follicular phase on the other hand did not seem to be suitable (*SMD* = −0.04). Taking a closer look at the hormone fluctuation and its consequences again, this finding is in accordance with Tenan et al. [47] regarding the luteal phase and its increased firing rate of motor units of the medial and oblique vastus, which increases the performance of explosive, maximal strength movements.

Considering maximal dynamic strength, only three studies including 43 participants assessed this outcome, with the result that the late follicular phase (*SMD* = 0.14) might be more suitable for this performance than the early follicular phase and the mid-luteal phase (*SMD* = −0.05). In the late follicular phase, high estrogen levels and low progesterone levels were found to increase muscle protein synthesis [89], promote muscle hypertrophy and muscle mass recovery [90], and increase type-II muscle fibers [91], which supports the findings of this meta-analysis. However, Thompson et al. [92] found that despite increased strength levels, explosive performance in the form of jumping and hopping decreases in the late follicular phase. The authors attributed this to other muscle and tendon characteristics such as reduced musculotendinous stiffness or increased joint laxity, which are associated with increased estrogen and decreased progesterone concentrations. Furthermore, the results regarding improved maximal strength in the late follicular phase partially contradict the most recent meta-analysis by Blagrove et al. [56], who found no differences related to the menstrual cycle phase. However, it should be noted that the authors of this study used a different phase model and only compared the early follicular, ovulatory, and mid-luteal phases, which means that differences in the late follicular phase were not taken into account.

It must be stated that the execution during the outcome measuring varied across the studies, for example regarding verbal encouragement or breaks between the attempts (20–180 s), which also might influence the results. Additionally, the participants were quite different and ranged from sedentary women to high-level athletes. This aspect should not be neglected, especially when it comes to psychological factors such as motivation, which is also influenced by the menstrual cycle [38] and is highly relevant for peak performance in sports. High-level athletes could be at an advantage here due to their experience and familiarity with testing situations.

Another strong point of criticism is the lack of blood samples to verify the menstrual cycle phases, which is the case in over half of the studies (54.5%). Furthermore, Allen et al. [93] showed that although the determination of cycle phases via blood samples is the most accurate, it also varies greatly depending on the frequency, timing, sensitivity, and type of analysis. These parameters appeared to be very heterogeneous within the studies. Although they all took measurements every morning [72,74,80,81,82,85,87], two were taken in the fasting state [75,78], two were non-fasted [72,80] and five did not specify further [74,81,82,85,87]. Six studies measured luteinizing (LH) and follicle-stimulating hormone (FSH) levels as well as estradiol and progesterone [72,74,75,80,81,82] levels, two only measured progesterone and estradiol levels [85,87], while one study only assessed LH and FSH levels for cycle phase determination [78]. The detection sensitivity (intra-assay / inter-assay coefficients of variation) ranged from 0.7 U/L (4.8%/9.4%) for LH, 0.1 U/L (5.4%/8.1%) for FSH, 2.9–55 pmol/L (5.2–10.6%/4.2–10.6%) for estradiol, and 0.16–0.6 nmol/L (4.1–10.3%/6.4–10%) for progesterone. However, four studies did not report on these measurement parameters [72,74,75,82].

In summary, the included studies are of low overall study quality due to the mostly imprecise determination of the cycle phases and should therefore be interpreted with caution. Out of twenty-two studies, fifteen achieved only low or very low study quality (68.2%) in the analysis using the adapted Downs and Black checklist, while six were moderate (27.3%), and only one of high quality (4.5%). As a result, the confidence in the evidence reported in the present systematic review with meta-analysis can be summarized as low. Thus, the results lose some level of meaning because the hormone levels present during the assessment are not clear. Comprehensive recommendations for action and comparisons between the studies become therefore unreliable. It is consequently mandatory to apply certain methodological requirements like menstrual cycle phase identification via blood samples, homogenous participant groups, outcomes, and consistent research designs to make studies more comparable as well as a priori sample size calculations to increase the study quality and therefore achieve significant and generalizable findings in future studies.

## 5. Conclusions

The current meta-analysis systematically evaluates the impact of hormone fluctuations during the menstrual cycle on maximal isometric, isokinetic, and dynamic strength in healthy women. Results show substantial variation between studies regarding the effects of hormone fluctuations on different maximal strength measures.

Overall, it can be concluded that the early follicular phase is unfavorable for all three tested maximal strength classes during the menstrual cycle. The late follicular phase is the optimal time for peak performance in isometric maximal strength, while the late luteal phase, likely due to low estrogen levels, is the least suitable. In contrast, isokinetic maximal strength is low during the late follicular phase, while the ovulatory phase is the best time according to the included studies, probably due to the increase in luteinizing hormone, which also stimulates testosterone availability. The late follicular phase appears to be the most conducive time for achieving dynamic maximal strength, possibly because of the high levels of estrogen present. Conversely, the mid-luteal phase is considered the least suitable time for optimal muscle function, due to decreased levels of estrogen (reduced neuromuscular facilitation) and increased levels of progesterone (decreased neuromuscular/synaptic transmission). This hormonal shift, as identified by Tenan et al. [47], suggests a potential reduction in maximal force generation during this phase, further influenced by the complex interplay with luteinizing hormone and its role in testosterone production.

The large variance among studies may be due to several methodological factors, the limited number of participants (Ø *n* = 19.7), and the associated high sample variance. Characteristics of participants, including sports history and related experience, may have also contributed to the significant heterogeneity observed between studies. On average, the studies investigated three conditions and involved fewer than 20 participants, leading to small effects.

The query arises as to who would benefit from discerning the nuanced variation in maximal strength performance during the menstrual cycle. In this regard, female competitive athletes are particularly pertinent. Thus, future studies should consider them as a distinct target population by incorporating more uniform participants to enhance result comparability. This review is the first to examine and identify differences in the three categories of maximal strength. However, none of the included studies examined the difference between all three categories, which could be a focus for future studies. Further attention should also be devoted to achieving high study quality, including implementing a suitable method for determining the correct menstrual cycle phase and conducting an a priori power analysis to optimize effect sizes and increase the informative value of the results. In particular, the late follicular phase and measurements of maximal dynamic strength should be given more attention in future studies. Due to the advantages in the hormonal regulation of this phase, especially during explosive movements, a transfer to sports-specific applications could be possible. The primary goal should be to obtain meaningful results that can inform practical recommendations, potentially also including those relevant to amateur athletes.

## Figures and Tables

**Table 1:** Overview of the applied inclusion and exclusion criteria.

Category	Inclusion Criteria	Exclusion Criteria
Population	women with a regular menstrual cycle	women taking hormonal contraceptives or who had menstrual cycle disorders
Intervention	assessment of maximal muscle strength in a clearly defined cycle phase	no measurements of maximal muscle strength or unclearly defined cycle phase
Comparator	early follicular phase	studies not including early follicular phase
Outcome	maximal isometric, isokinetic, or dynamic muscle strength	muscle power or impulse
Study design	longitudinal	between-subjects design, case studies, reviews

**Table 2:** Overview of the preferred and alternative outcome by category.

Category	Preferred Outcome	Alternative Outcome
Isometric maximal strength	MVIC knee extension (*n* = 7)	MVIC handgrip strength (*n* = 5) MVIC FDI strength (*n* = 1) MVIC leg press (*n* = 1) MVIC shoulder internal rotation (*n* = 1)
Isokinetic maximal strength	Peak torque knee extension 60°/s (*n* = 4)	Peak torque knee extension 120°/s (*n* = 2) Peak torque knee extension 90°/s (*n* = 1) Peak torque knee extension 30°/s (*n* = 1)
Dynamic maximal strength	1RM half-squat (*n* = 1)	1RM leg press (*n* = 1) 1RM bench press (*n* = 1)

MVIC = maximal voluntary isometric contraction; 1RM = one repetition maximum; FDI = first dorsal interosseus muscle.

**Table 3:** The influence of menstrual cycle phases on maximal strength performance (isometric strength/isokinetic strength/dynamic strength).

References	No. of Subjects (Sex); Activity; Age (Mean ± SD, or Range); Height (Mean ± SD, or Range); Mass (Mean ± SD, or Range); Determination of Cycle Phase	Compared Menstrual Cycle Phases	Muscle Groups or Function Examined; Method; Side	Test Modality; Outcome Measures	Results, Mean (SD)	Level of Evidence
Arazi et al. [78]	20 (F); recreationally active; 26.27 ± 2.75 yrs; 161 ± 4.25 cm; 60.14 ± 7.60 kg; blood sample	COMP1: ovulation (days 12–14) COMP2: mid-luteal (days 18–25) CON: early follicular (days 3–8)	Leg press; dynamic strength; bilateral	Calculation: 1RM with repetitions until exhaustion	**1RM leg press (kg)** COMP1: 72 (5.72) COMP2: 74 (4.66) CON: 75 (3.25)	Low
Bambaeichi et al. [71]	8; sedentary/inactive; 30 ± 5 yrs; 163 ± 6 cm; 66.25 ± 4.6 kg; urine sample	COMP1: late follicular (days 7–9) COMP2: ovulation COMP3: mid-luteal (days 19–21) CON: early follicular (days 1–4)	Knee extension; isokinetic strength; dominant	1 familiarization trial Warm-up: 5 min cycle ergometer (80–120 W) 3 attempts; 180 s rest between; supine position; knee ROM 90°	**Peak torque knee extension at 90°/s (Nm)** COMP1: 72.1 (5) COMP2: 76.8 (5.7) COMP3: 76.9 (5) CON: 73.2 (5)	Low
Dasa et al. [72]	8; high-level athletes; 22.5 ± 4.2 yrs; 168.8 ± 8.9 cm; 63.1 ± 9.3 kg; blood sample	COMP1: late follicular (week 2) COMP2: early luteal (week 3) COMP3: mid-luteal (week 4) CON: early follicular (week 1)	Handgrip; isometric strength; dominant	Warm-up: 15 min cycle ergometer (100 W) Handgrip: 2 attempts; seated; 90° elbow flexion; 3–5 s duration; 30 s rest between	**MVIC handgrip strength (kg)** COMP1: 25.8 (2.9) COMP2: 28.7 (3.6) COMP3: 25.6 (2.9) CON: 25.1 (4.9)	Moderate
Dibrezzo et al. [79]	21; healthy; 18–36 yrs; NA; 65 ± 9 kg; counting days	COMP1: ovulation (days 13–14) COMP2: mid-luteal (day 10 from ovulation) CON: early follicular (day 1)	Knee flexion and extension; isokinetic strength; dominant	3 familiarization trials 4 attempts; 45 s rest between; velocity 60°/s and 180°/s	**Peak torque knee extension at 60°/s (Nm)** COMP1: 138.3 (21.8) COMP2: 134.8 (17.7) CON: 137.4 (24.3)	Very low
Drake et al. [73]	13; inactive; 24 ± 1 yrs; 1.68 ± 0.02 m; 70.97 ± 4.81 kg; urine sample	COMP1: late follicular (days 9–11) COMP2: ovulation COMP3: early luteal (5 days after ovulation) CON: early follicular (days 1–3)	Knee extension; isometric strength; dominant	1 familiarization trial 2 max attempts; highest used for analysis; 3 s duration; 180 s rest between; 45° leg flexion	**MVIC knee extension (Nm)** COMP1: 139.3 (15.5) COMP2: 139.3 (14.4) COMP3: 134.6 (13.9) CON: 136.4 (14.5)	Very low
Ekenros et al. [80]	9; recreationally active; 27.0 ± 4.8 yrs; 166.8 ± 5.2 cm; 61.8 ± 6.5 kg; blood sample	COMP1: ovulation COMP2: mid-luteal (days 7–8 after ovulation) CON: early follicular (days 2–4)	Hand grip; isometric strength; dominant Knee extension; isokinetic strength; right	3 familiarization trials Warm-up: 10 min cycle ergometer (150 W) Handgrip strength: 3 attempts; elbow extended, arm beside body Knee extension: 5 attempts; back supporter in 85° angle; velocity 120°/s; knee ROM 90–10°	**MVIC handgrip strength (kg)** COMP1: 28 (4) COMP2: 28 (4) CON: 28 (4) **Peak torque knee extension 120°/s (Nm)** COMP1: 141 (26) COMP2: 145 (26) CON: 139 (28)	Moderate
Elliot et al. [86]	7; healthy; 25 ± 5 yrs; 1.6 ± 0.1 m; 62.1 ± 2.7 kg; urine sample	INT: mid-luteal (day 21) CON: early follicular (day 2)	First dorsal interosseus muscle; isometric strength; dominant	2 familiarization trials Warm-up: hand and forearm with heating pad to 40 °C skin temperature 3 sub-max attempts; 180 s rest between; 3 max attempts for data; 60 s rest between	**MVIC FDI strength (N)** INT: 30.8 (7.4) CON: 28.2 (4.3)	Low
Farouzandeh Sharaki et al. [65]	15; athletes; 23.27 ± 1.66 yrs; 167 ± 0.05 cm; 57.60 ± 6.75 kg; urine sample	COMP1: ovulation (days 1–2 after ovulation) COMP2: mid-luteal (day 7 after ovulation) CON: early follicular (days 1–3)	Shoulder internal rotation; isometric strength; dominant	90° angle in the elbow Internal rotation in the supine position	**MVIC shoulder internal rotation (kg)** COMP1: 10.57 (1.69) COMP2: 9.06 (1.62) CON: 9.33 (1.84)	Low
Fridén et al. [81]	10; recreationally active; 25.3 ± 3.7 yrs; 171 ± 5 cm; 66 ± 7 kg; blood sample	COMP1: ovulation COMP2: mid-luteal (day 7 after ovulation) CON: early follicular (days 3–5)	Handgrip; isometric strength; dominant Knee extension; isokinetic strength; right	1 familiarization trial Warm-up: 10 min cycle ergometer Handgrip: 3 attempts; elbow extended, arm beside body Knee extension: 5 attempts; back supporter in 85° angle; velocity 120°/s; knee ROM 90–10°	**MVIC handgrip strength (kg)** COMP1: 37 (6) COMP2: 37 (8) CON: 36 (6) **Peak torque knee extension 120°/s (Nm)** COMP1: 147 (20) COMP2: 146 (22) CON: 145 (24)	Moderate
Gordon et al. [31]	11; athletes; 20.7 ± 1.4 yrs; 166.8 ± 7.1 cm; 59.2 ± 6.9 kg; saliva sample	COMP1: late follicular (days 9–11) COMP2: mid-luteal (days 19–20) COMP3: late luteal (days 27–28) CON: early follicular (days 1–3)	Knee extension; isokinetic strength; NA	Warm-up: 4 concentric extension/flexions at 60°/s, followed by a 30 s recovery period 5 attempts; 180 s rest between; velocity 60°/s	**Peak torque knee extension at 60°/s(Nm)** COMP1: 116.8 (23.63) COMP2: 116.8 (22.49) COMP3: 116.2 (22.34) CON: 110.7 (25.23)	Low
Hoshi [74]	14; healthy; 19.6 ± 1 yrs; 162.1 ± 4.3 cm; 46.2 ± 5.5 kg; blood sample	COMP1: late follicular (days 7–12) COMP2: ovulation (days 1–2 after start ovulation) COMP3: mid-luteal (days 7–12 after start of ovulation) CON: early follicular (days 2–5)	Handgrip; isometric strength; NA	No information provided	**MVIC handgrip strength (kg)** COMP1: 39.2 (6.1) COMP2: 38.4 (5.1) COMP3: 38.2 (6.3) CON: 36.3 (5.1)	Low
Janse de Jonge et al. [75]	15; healthy; 29.9 ± 8.0 yrs; 167 ± 7 cm; 61.4 ± 8.4 kg; blood sample	COMP1: late follicular COMP2: mid-luteal CON: early follicular (days 1–3)	Knee flexion and extension; isometric and isokinetic strength; dominant	Warm-up: 10 min cycle ergometer (50 W) with cadence of 60 rpm Knee extension isometric: 5 attempts; 120 s rest between Knee flexion isokinetic: 5 attempts; 120 s rest between; 60°/s and 240°/s Knee extension isokinetic: 5 attempts; 120 s rest between; 60°/s and 240°/s	**MVIC knee extension (N)** COMP1: 551 (114) COMP2: 570 (109) CON: 571 (114) **Peak torque knee extension at 60°/s (Nm)** COMP1: 148.9 (27.7) COMP2: 152.8 (27.8) CON: 153.6 (33.0)	Moderate
Koese [76]	10; athletes; 21.4 ± 2.01 yrs; 169.6 ± 6.14 cm; 63.9 ± 5.76 kg; counting days	COMP1: late follicular (days 8–9) COMP2: mid-luteal (days 22–23) CON: early follicular (days 2–3)	Bench press; dynamic strength; bilateral	1RM test (until exhaustion with 65% of 1RM)	**1RM bench press (kg)** COMP1: 41.7 (6.4) COMP2: 41.65 (6.72) CON: 41.2 (6.54)	Very low
Kubo et al. [82]	8; sedentary; 22.5 ± 0.9 yrs; 160.2 ± 5.1 cm; 56.4 ± 3.7 kg; blood sample	COMP1: ovulation (days 1–2 after ovulation) COMP2: mid-luteal (days 7–10 after ovulation) CON: early follicular (days 1–3)	Knee extension; isometric strength; right	Warm-up: dynamic submaximal knee extension 3 attempts; 5 s duration; 180 s rest between; 90° knee flexion; strong verbal encouragement	**MVIC knee extension (Nm)** COMP1: 112.3 (22.7) COMP2: 113.6 (28.6) CON: 113.9 (21.6)	Moderate
Lebrun et al. [87]	16; athletes; 27.6 ± 3.8 yrs; 167.9 ± 5.3 cm; 59.6 ± 6.7 kg; blood sample	INT: mid-luteal (days 4–9 after ovulation) CON: early follicular (days 3–8)	Knee flexion and extension; isokinetic; right	Warm-up: short and unspecific 3 attempts; seated	**Peak torque knee extension at 30°/s (Nm)** INT: 142.5 (6.2) CON: 143.9 (7.9)	Moderate
Miyazaki and Maeda [83]	16; healthy; 21 ± 1 yrs; 159.5 ± 4.7 cm; 52.5 ± 5 kg; urine sample	COMP1: ovulation (days 2–3 after ovulation) COMP2: mid-luteal (days 20–22) CON: early follicular (day 3)	Knee extension; isometric strength; right	1 attempt; 3 s duration; prone position; 90° knee flexion; verbal encouragement	**MVIC knee extension (Nm)** COMP1: 35.3 (8.2) COMP2: 39 (5.8) CON: 38.9 (9.9)	Low
Montgomery & Shultz [85]	61; recreationally active; COMP1: 21.5 ± 2.7 yrs; 164.9 ± 7.2 cm; 61.7 ± 9.3 kg; Blood sample COMP2: 21.2 ± 2.4 yrs; 164.0 ± 6.6 cm; 60.8 ± 9.1 kg; blood sample	COMP1 (*n* = 29): Early luteal (days 10–24) CON1 (*n* = 29): Early follicular (days 1–3) COMP2 (*n* = 32): Mid-luteal (days 15–28) CON2 (*n* = 32): Early follicular (days 1–3)	Knee flexion and extension; isometric strength; dominant	Familiarization trials at 25, 50, 75, and 100% perceived maximal effort Warm-up: 5 min cycle ergometer 3 attempts; 3 s duration; 30 s rest between; 20° knee flexion; strong verbal encouragement	**MVIC knee extension (Nm/kg)** COMP1: 2.3 (0.5) CON1: 2.4 (0.5) COMP2: 2.2 (0.4) CON2: 2.3 (0.4)	High
Pallavi et al. [32]	100; healthy; 18.4 ± 0.7 yrs; 150 ± 6 cm; 50 ± 4.9 kg; NA	COMP1: late follicular COMP2: mid-luteal CON: Early follicular (day 1)	Handgrip; isometric strength	3 attempts; verbal encouragement	**MVIC handgrip strength (kg)** COMP1: 33.04 (3.7) COMP2: 27.3 (3.4) CON: 22.5 (3.4)	Very low
Poumasiri et al. [84]	37; athletes; 21.65 ± 3.5 yrs; 171.14 ± 9.2 cm; 64.07 ± 4.9 kg; self-report	COMP1: ovulation (days 10–14) COMP2: mid-luteal (days 15–28) CON: early follicular (days 1–9)	Knee flexion and extension; isokinetic and isometric strength; dominant	Warm-up: 15 min cycle ergometer 3 attempts; 20 s rest between; back supporter in 70–85° angle; velocity 60°/s; knee ROM 0–90°	**Peak torque knee extension at 60°/s (Nm)** COMP1: 131.8 (21.1 COMP2: 116.6 (9.7) CON: 115.4 (18.8) **MVIC knee extension (Nm^2^)** COMP1: 65.1 (9.6) COMP2: 51.8 (8.4) CON: 52.7 (8.9)	Very low
Rodrigues et al. [77]	12; recreationally active; 27.2 ± 3.4 yrs; 168.62 ± 5.19 cm; 61.37 ± 6.29 kg; counting days	COMP1: late follicular (days 2–3) COMP2: late luteal (days 2–3 before menstruation) CON: early follicular (days 1–2 of menstruation)	Leg press; isometric strength; bilateral	1 familiarization trial Warm-up: 5 repetitions of leg press with 50% and then 70% 1RM with 2 min set break 3 attempts; 180 s rest between; 45° knee flexion (2–10 reps, with increments of 2, 3 or 4% with more than 10 possible reps	**MVIC leg press (kg)** COMP1: 248.26 (48.39) COMP2: 220.97 (46.63) CON: 237.89 (50.19)	Very low
Romero-Moraleda et al. [6]	13; athletes; 31.1 ± 5.5 yrs; 166 ± 6 cm; 58.6 ± 7.8 kg; urine sample	COMP1: late follicular (days 8–14) COMP2: mid-luteal (days 15–28) CON: early follicular (days 1–7)	Half squat; dynamic strength; bilateral	1 familiarization trial 1RM test with loads to 20, 40, 60, and 80% of 1RM, vertical guides regulate the barbell movement	**1RM half-squat (kg)** COMP1: 98.5 (18.1) COMP2: 98.1 (22.2) CON: 97 (23.2)	Low
Tenan et al. [28]	9; recreationally active; 24.7 ± 4.5 yrs; body temperature	COMP1: late follicular (days 7–12) COMP2: ovulation (days 13–15) COMP3: early luteal (days 16–22) COMP4: late luteal (days 22–28) CON: early follicular (days 1–6)	Knee extension; isometric strength; dominant	Warm-up: 12 dynamic submaximal knee extension without resistance 3 attempts; 3 s duration; 30 s rest between; seated; 90° knee flexion; strong verbal encouragement	**MVIC knee extension (kg)** COMP1: 45.0 (8.48) COMP2: 44.87 (8.69) COMP3: 37.3 (8.7) COMP4: 45.68 (8.79) CON: 43.2 (8.45)	Very low

COMP = comparator condition (i.e., at least one other cycle phase like late follicular phase, etc.); CON = control condition (i.e., early follicular phase); MVIC = maximal voluntary isometric contraction; FDI = first dorsal interosseus muscle; 1RM = one repetition maximum; REP = repetition; rpm = rounds per minute.

## Data Availability

The original contributions presented in the study are included in the article/Supplementary Materials, further inquiries can be directed to the corresponding author.

## Supplementary Materials

The following supporting information can be downloaded at: https://www.mdpi.com/article/10.3390/sports12010031/s1, Table S1: PRISMA checklist: Transparent reporting of systematic reviews and meta-analyses; Table S2: Modified Downs and Black checklist.
