# Supplementary material for: The Influence of Menstrual Cycle Phases on Maximal Strength Performance in Healthy Female Adults: A Systematic Review with Meta-Analysis
Source: Sports (Basel). 2024 Jan 12;12(1):31. doi: 10.3390/sports12010031 (PMC10818650; doi:10.3390/sports12010031)
Supplement: Supplementary file 1 [file sports-12-00031-s001.zip › Table S2.pdf]

sports

MDPI

Table S2. Modified Downs and Black checklist according to McNulty et al. (2020)

	Arazi et al. (2018)	Bambaeichi et al. (2009)	Dasa et al. (2021)	Dibrezzo et al. (1988)	Drake et al. (2003)	Ekenros et al. (2013)	Elliot et al. (2003)	Farouzandeh Sharaki et al. (2020)	Fridén et al. (2003)	Gordon et al. (2013)	Hoshi (1996)	Janse de Jonge et al. (2001)	Koese (2018)	Kubo et al. (2009)	Lebrun et al. (1995)	Miyazaki et al. (2022)	Montgomery & Shultz (2010)	Pallavi et al. (2017)	Poumasiri et al. (2023)	Rodrigues et al. (2019)	Romero-Moraleda et al. (2019)	Tenan et al. (2016)
Q1. Is the hypothesis/aim/objective of the study clearly described?<br>Yes = 1 / No = 0	1	1	1	1	1	1	1	1	1	1	1	1	1	1	1	1	1	0	1	1	1	1
Q2. Are the main outcomes to be measured clearly described in the introduction or methods section? If the main outcomes are first mentioned in the results section, answer no.<br>Yes = 1 / No = 0	0	0	0	0	1	1	1	1	0	1	0	1	0	1	1	1	1	0	0	0	0	0
Q3. Are the characteristics of the participants included in the study clearly described?<br>Yes = 1 / No = 0 / Unable to determine = 0	1	1	1	1	1	1	1	1	1	1	1	1	1	1	1	1	1	1	1	1	1	1

Q4. Were the tested menstrual cycle phases clearly described? Answer yes if the precise criteria used to define phase were provided, answer no if the exact phase tested cannot be ascertained (e.g., vague language such as "early" or "late" were used, without defining the criteria)<br>Yes = 1 / No = 0	1	1	1	1	1	1	1	1	1	1	1	1	1	1	1	1	1	1	1	1	1	1
Q5. Are the main findings of the study clearly described? Simple outcome data should be reported for all major findings so the reader can check the major analyses and conclusions. This does not cover statistical tests which are addressed in other questions.<br>Yes = 1 / No = 0	1	1	1	1	1	1	1	1	1	1	1	1	1	1	1	1	1	1	1	1	1	1
Q6. Does the study provide estimates of the random variability in the data for the main outcomes? In non-normal data, interquartile range should be reported. In normal data, standard deviation, standard error or confidence intervals should be reported.<br>Yes = 1 / No = 0	1	1	1	1	1	1	1	1	1	1	1	1	1	1	1	1	1	1	1	1	1	1
Q7. Were the subjects confirmed as non-hormonal contraceptive users, for at least three months prior to participation?<br>Yes = 1 / No = 0 / Unable to determine = 0	1	1	1	1	0	1	1	1	1	0	0	1	1	1	0	1	1	0	0	1	1	1
Q8. Was at least one familiarization trial conducted prior to exercise testing?<br>Yes = 1 / No = 0 / Unable to determine = 0	0	1	0	1	1	1	1	0	1	1	0	0	0	1	1	0	1	0	0	1	1	0
Q9. Were the exercise test conditions adequately standardised (taking into consideration factors including time of day, prior nutritional intake [including caffeine] and prior exercise).<br>Yes (all relevant factors) = 2 / Yes (some relevant factors) = 1 / Exercise testing unstandardized = 0 / Unable to determine = 0	0	1	1	1	0	1	2	1	1	1	0	0	1	1	1	1	1	1	1	1	1	2

Q10. If any of the results of the study were based on 'data dredging' was this made clear? Any analyses that had not been planned at the outset should be clearly indicated. If no retrospective subgroup analyses were reported, then answer yes.<br>Yes = 1 / No = 0 / Unable to determine = 0	0	1	0	1	1	1	1	1	1	1	1	1	1	1	1	1	1	1	0	0	1	1
Q11. Were statistical tests used to assess the main outcomes appropriate? The statistical techniques used must be appropriate to the data and the research question.<br>Yes = 1 / No = 0 / Unable to determine = 0	1	1	1	0	1	1	1	1	1	1	0	1	1	1	1	1	1	1	1	1	1	1
Q12. Were the main outcome measures used accurate (i.e., valid and reproducible)? For studies where the validity and reproducibility of outcome measures are clearly described, the question should be answered yes. For studies which refer to other work that demonstrates the outcome measures are accurate, answer yes.<br>Yes = 1 / No = 0 / Unable to determine = 0	0	1	1	0	0	1	0	0	1	1	0	1	1	1	1	1	1	0	0	1	1	0
Q13. Was the order of phase testing randomised?<br>Yes = 1 / No = 0 / Unable to determine = 0	1	1	1	0	0	0	1	1	1	1	1	0	1	1	1	1	1	1	0	1	1	1
Q14. Did the study have sufficient power to detect an a priori specified scientifically important effect at a pre-determined probability threshold? Answer yes if they included a power calculation, and no if not.<br>Yes = 1 / No = 0	0	0	0	0	0	1	0	0	0	0	0	0	0	0	1	1	0	0	0	0	0	0
Q15. Was study retention > 85%?<br>Yes = 1 / No = 0 / Unable to determine = 0	0	0	0	0	0	0	0	0	0	0	0	0	0	0	0	0	1	0	0	1	0	0
Q16. Identify if menstrual cycle phase was confirmed using blood samples. If yes, the a priori rating is maintained and this is the final study quality rating. If not, the study is downgraded a level (e.g., a study that started out as high, drops to moderate).	Yes	No	Yes	No	No	Yes	No	No	Yes	No	Yes	Yes	No	Yes	Yes	No	Yes	No	No	No	No	No

Q17. Identify if menstrual cycle phase was confirmed using ovulation kits. If yes, the Q1. rating is maintained. If no, the study is downgraded another level (e.g., a study that started out high, drops to low). This means that the maximum rating that any study that does not use blood analysis or ovulation kits is "low" or "very low".	Yes	Yes	Yes	No	Yes	Yes	Yes	Yes	Yes	Yes	Yes	Yes	No	Yes	Yes	Yes	Yes	No	No	No	Yes	No
**Rating**	Maintain	1 Downgrade	Maintain	2 Downgrades	1 Downgrade	Maintain	1 Downgrade	1 Downgrade	Maintain	1 Downgrade	Maintain	Maintain	2 Downgrade	Maintain	Maintain	1 Downgrade	Maintain	2 Downgrades	2 Downgrades	2 Downgrades	1 Downgrade	2 Downgrades
A priori	low	moderate	moderate	low	low	moderate	Moderate	moderate	moderate	moderate	low	moderate	moderate	moderate	moderate	moderate	high	low	low	moderate	moderate	moderate
**Final classification**	low	low	moderate	very low	very low	moderate	low	low	moderate	low	low	moderate	very low	moderate	moderate	low	high	very low	very low	very low	low	very low
